# Association Between Surgeons’ Consideration of Suspected Biliary Contamination and Reported Antibiotic Duration After Pancreatoduodenectomy: An Exploratory Secondary Analysis of an International Survey

**DOI:** 10.3390/antibiotics15090922

**Published:** 2026-09-17

**Authors:** Michael Hoffmann, David Farkas, Florian Schepp, Christina A. Neizert, Franz G. M. Poch, Katharina Beyer, David Pinto

**Affiliations:** Department of General, Visceral, Transplant and Thoracic Surgery, University Hospital Augsburg, Stenglinstrasse 2, 86156 Augsburg, Germany; david.farkas@uk-augsburg.de (D.F.); florian.schepp@uk-augsburg.de (F.S.); christina.neizert@uk-augsburg.de (C.A.N.); franz.poch@uk-augsburg.de (F.G.M.P.); katharina.beyer@uk-augsburg.de (K.B.);

**Keywords:** pancreatoduodenectomy, postoperative pancreatic fistula, biliary contamination, antibiotic prophylaxis, antimicrobial stewardship, perioperative antibiotics, surgical site infection

## Abstract

**Background/Objectives:** Biliary contamination has been linked to infectious complications and clinically relevant postoperative pancreatic fistula (CR-POPF) after pancreatoduodenectomy (PD). It remains unclear whether surgeons consider suspected biliary contamination when assessing POPF risk and whether this is reflected in the antibiotic management they report. **Methods:** This exploratory secondary analysis examined responses from an international survey endorsed by the E-AHPBA. Associations between consideration of suspected biliary contamination and reported antibiotic class selection, routine duration category, and adaptation of duration in cases of suspected or proven biliary contamination were analyzed using Fisher’s exact tests and ordinal logistic regression. **Results:** Among 283 respondents, 28 (9.9%) reported considering suspected biliary contamination when assessing POPF risk. Of 268 surgeons reporting perioperative antibiotic use, 185 (69.0%) reported adapting antibiotic duration in this setting. Surgeons who considered suspected biliary contamination reported longer antibiotic duration (OR 2.29, 95% CI 1.14–4.59; *p* = 0.020) and more often reported adapting duration (OR 4.15, 95% CI 1.21–22.11; *p* = 0.016). No statistically significant difference in reported antibiotic class selection was detected (global *p* = 0.192), including for ureidopenicillin use (OR 1.48, 95% CI 0.63–3.55; *p* = 0.422). **Conclusions:** In this international survey, few surgeons reported considering suspected biliary contamination when assessing POPF risk. Those who did reported longer routine antibiotic duration and more frequently reported adapting duration in cases of suspected or proven biliary contamination. Differences in reported antibiotic class selection remained uncertain. These findings are relevant from an antimicrobial stewardship perspective, as current evidence provides stronger support for covering the expected biliary pathogens than for prolonging prophylaxis, particularly in patients with preoperative biliary drainage.

## 1. Introduction

Postoperative infectious complications remain common after pancreatoduodenectomy (PD) and are a major source of morbidity [1,2,3]. Clinically relevant postoperative pancreatic fistula (CR-POPF), as defined by the International Study Group of Pancreatic Surgery [4], is a pancreatic leak that alters the postoperative course and requires a change in management. It is defined by its clinical impact rather than by infection itself, although infection may contribute to progression of the leak. Patients with CR-POPF often require prolonged drainage, antibiotics, radiological or surgical intervention, and have delayed recovery. Although pancreatic texture, duct size, and BMI remain central components of established POPF risk models, increasing evidence suggests that microbiological factors may also influence whether a postoperative pancreatic leak remains clinically silent or meets criteria for CR-POPF [5,6,7,8].

Biliary contamination is of particular interest. Patients with preoperative biliary drainage often have contaminated bile, including organisms that are resistant to standard prophylaxis. Recent randomized and observational data have linked biliary contamination, antimicrobial resistance, and both infectious complications and clinically relevant POPF after PD, particularly in patients with preoperative biliary drainage [9,10,11]. These findings suggest that antimicrobial coverage of biliary organisms may directly influence fistula-related morbidity.

It is unclear whether this evidence has influenced how surgeons assess risk and choose antibiotics in daily practice. In particular, it is unknown whether suspected biliary contamination is treated as a POPF-related risk factor, and whether this leads surgeons to choose a different antibiotic class, extend antibiotic duration, or adapt antibiotic duration in response to biliary contamination.

An international E-AHPBA–endorsed survey previously described contemporary POPF-prevention practices after PD, including risk assessment and perioperative antibiotic strategies [12]. However, the primary report did not examine whether consideration of suspected biliary contamination in POPF risk assessment was associated with specific antibiotic-management decisions. We therefore investigated whether surgeons who consider suspected biliary contamination report a different antibiotic class, a different routine duration, or adaptation of duration. We hypothesized that few surgeons would consider suspected biliary contamination when assessing POPF risk, and that those who did would report a longer duration more consistently than a different antibiotic class.

## 2. Results

### 2.1. Respondents

Of 283 respondents who completed the survey, 268 (94.7%) reported administering perioperative antibiotics and were included in the antibiotic strategy analyses.

Among all 283 respondents, only 28 (9.9%) reported considering suspected biliary contamination when assessing POPF risk. Among the 268 respondents who reported administering perioperative antibiotics, 185 (69.0%) reported adapting antibiotic duration in cases of suspected or proven biliary contamination.

### 2.2. Respondent Characteristics

Table 1 shows respondent characteristics by contamination in POPF risk assessment. Consideration was not significantly associated with institution type, institutional PD volume, personal annual PD volume, years in practice, or self-reported risk-adapted management.

Self-reported risk-adapted management was common in both groups. It was reported by 193 of 255 surgeons (75.7%) who did not consider suspected biliary contamination and by 20 of 28 surgeons (71.4%) who did consider it (*p* = 0.646).

### 2.3. Reported Antibiotic Class Selection (AP02)

**Figure 1:**
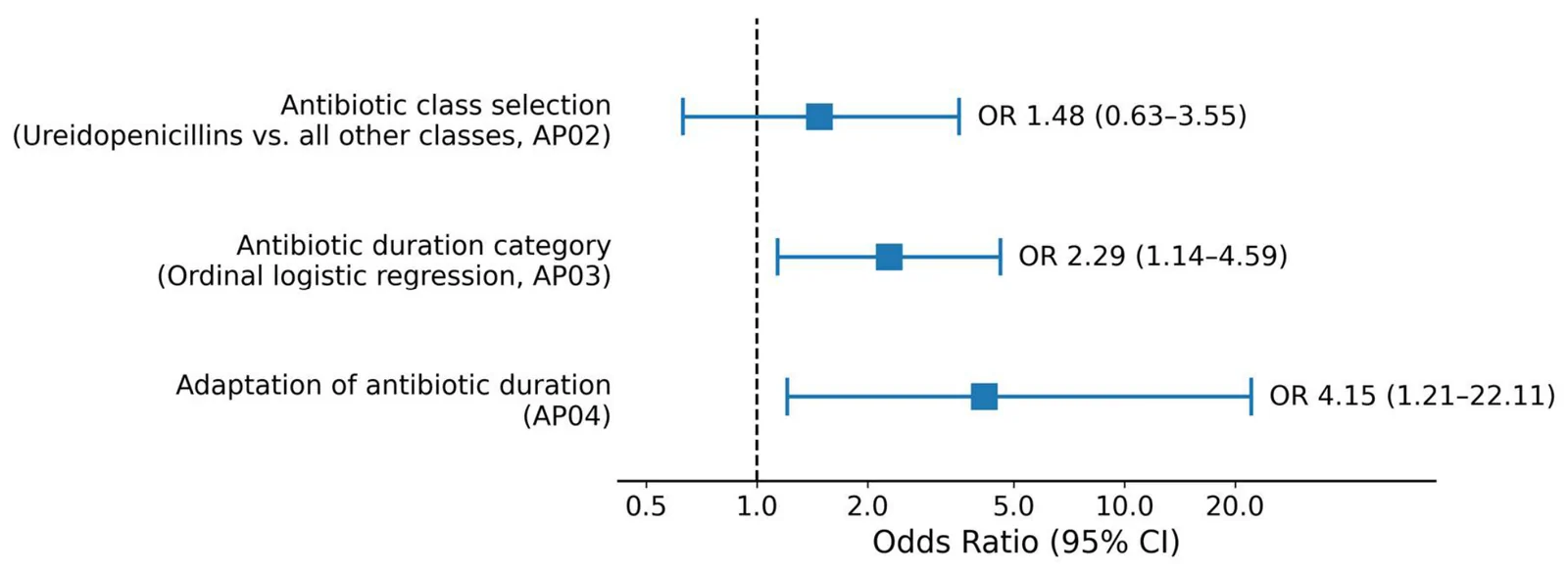
Association between consideration of suspected biliary contamination in POPF risk assessment and reported perioperative antibiotic decisions. Squares indicate the odds ratios and horizontal lines the corresponding 95% confidence intervals, shown for antibiotic class selection (ureidopenicillins vs. all other antibiotic classes, AP02), antibiotic duration category (ordinal logistic regression, AP03), and antibiotic duration adaptation (AP04). The dashed vertical line indicates the null value (OR = 1); estimates to its right indicate higher odds in surgeons who considered suspected biliary contamination. Analyses are restricted to the 268 respondents reporting perioperative antibiotic use.

**Figure 2:**
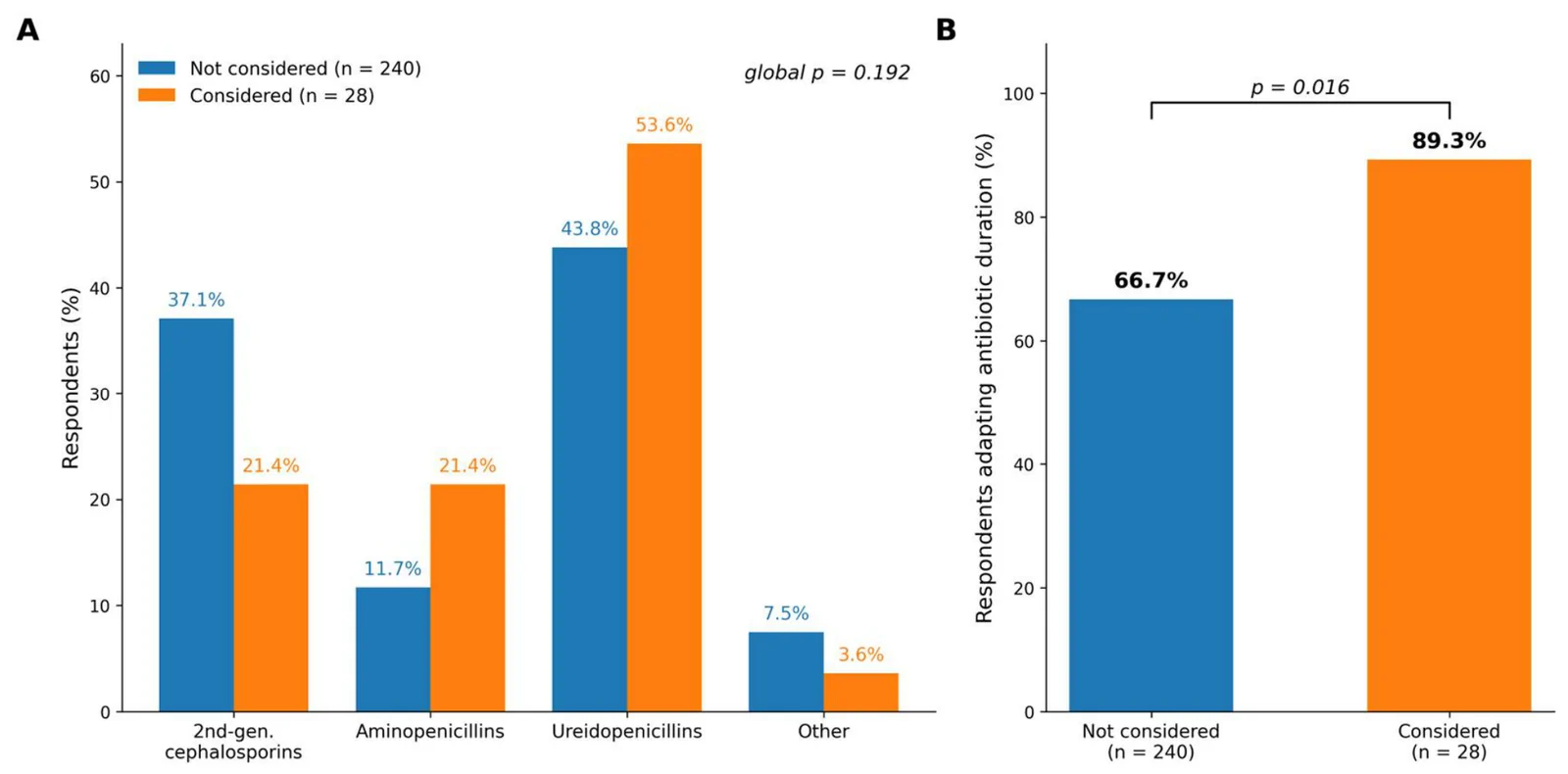
Reported antibiotic decisions according to consideration of suspected biliary contamination in POPF risk assessment. (**A**) Distribution of antibiotic classes according to whether suspected biliary contamination was considered in risk assessment; no statistically significant difference in antibiotic class selection was detected (global *p* = 0.192). (**B**) Proportion of surgeons who indicated that they would adapt antibiotic duration; adaptation was more frequently reported among those who considered suspected biliary contamination (89.3% vs. 66.7%; OR 4.15, 95% CI 1.21–22.11; *p* = 0.016).

Among the 268 respondents administering perioperative antibiotics, reported antibiotic class selection did not differ significantly between surgeons who did and did not consider suspected biliary contamination (global Fisher–Freeman–Halton *p* = 0.192; Table 2). Figure 1 summarizes the effect estimates for all three antibiotic outcomes, and Figure 2A shows the full distribution of antibiotic classes.

Ureidopenicillins were selected by 15 of 28 surgeons (53.6%) who considered suspected biliary contamination and by 105 of 240 surgeons (43.8%) who did not (absolute difference 9.8 percentage points, 95% CI −9.7 to 29.3; OR 1.48, 95% CI 0.63–3.55; *p* = 0.422). Six of 28 surgeons (21.4%) used second-generation cephalosporins when they suspected biliary contamination, and 89 of 240 (37.1%) did not. Aminopenicillins were used by 6 of 28 (21.4%) and 28 of 240 (11.7%), respectively, and other regimens by 1 of 28 (3.6%) and 18 of 240 (7.5%).

In an exploratory analysis, antibiotic class distribution did not differ significantly between respondents who did and did not report adapting of antibiotic duration (global Pearson χ^2^ *p* = 0.270; Table S1).

### 2.4. Reported Antibiotic Duration (AP03)

In contrast to antibiotic class selection, consideration of suspected biliary contamination was associated with longer antibiotic duration. In the ordinal logistic regression model, consideration was associated with higher odds of being in a longer duration category (OR 2.29, 95% CI 1.14–4.59; *p* = 0.020; Table 2, Figure 1). The proportional odds assumption was not rejected by the test of parallel lines (χ^2^ = 4.16, df = 2, *p* = 0.125).

Among surgeons who considered suspected biliary contamination, 15 of 28 (53.6%) reported antibiotic administration for 3–5 days, compared with 61 of 240 (25.4%) among those who did not. Intraoperative-only prophylaxis was less common among surgeons who considered suspected biliary contamination than among those who did not (25.0% vs. 47.5%).

Exploratory cumulative binary models were examined at all three duration thresholds. Consideration of suspected biliary contamination was associated with postoperative continuation versus intraoperative-only prophylaxis (OR 2.70, 95% CI 1.06–7.82; *p* = 0.027) and with antibiotic administration for ≥3 days versus <3 days (OR 2.81, 95% CI 1.18–6.86; *p* = 0.011). At the highest threshold, more than five days versus five days or less, the estimate was based on 1 of 28 and 16 of 240 respondents and was uninformative (OR 0.52, 95% CI 0.01–3.61; *p* = 1.000).

Adaptation also examined adaptation of antibiotic duration according to routine prophylaxis duration (Table S2). Among the 121 respondents using intraoperative-only prophylaxis, 74 (61.2%) indicated that they would adapt duration in cases of suspected or proven biliary contamination; in this subgroup, adaptation necessarily implies prolongation. These 74 respondents accounted for 40.0% of all respondents indicating adaptation.

### 2.5. Reported Adaptation of Antibiotic Duration (AP04)

Consideration of suspected biliary contamination was also associated with adaptation of antibiotic duration. Among surgeons who considered suspected biliary contamination in POPF risk assessment, 25 of 28 (89.3%) indicated that they would adapt antibiotic duration, compared with 160 of 240 (66.7%) among those who did not (absolute difference 22.6 percentage points, 95% CI 9.7 to 35.5; prevalence ratio 1.34, 95% CI 1.15–1.57; OR 4.15, 95% CI 1.21–22.11; *p* = 0.016; Table 2, Figure 2B).

Figure 1 summarizes the effect estimates across the main association analyses. Overall, considering suspected biliary contamination was associated with longer and more frequently adjusted antibiotic treatment, whereas the analysis did not provide clear evidence of an association with antibiotic class selection.

### 2.6. Sensitivity Analyses

Adjustment for geographic region and institutional pancreatoduodenectomy volume only modestly attenuated the association with reported routine antibiotic duration (aOR 2.09, 95% CI 1.03–4.25; Table S3). The estimate for duration adaptation remained similar (aOR 3.51, 95% CI 1.01–12.19). For ureidopenicillin use, the adjusted estimate remained imprecise and compatible with no clear difference (aOR 1.86, 95% CI 0.82–4.19). Adjustment for geographic region alone did not materially change any of the estimates.

After Benjamini–Hochberg correction across the three principal antibiotic outcomes, the associations with reported routine duration (q = 0.030) and adaptation of duration (q = 0.030) remained below the 0.05 false-discovery-rate threshold, whereas the global antibiotic class-selection comparison remained non-significant (q = 0.192).

## 3. Discussion

In this secondary analysis of an international survey, fewer than 10% of respondents reported considering biliary contamination in POPF risk assessment. Those who did reported longer routine antibiotic courses and more often adjusted antibiotic duration. In contrast, differences in reported antibiotic class selection, including ureidopenicillin use, remained uncertain. The association was therefore clearer for reported antibiotic duration than for reported antibiotic class selection. This is clinically relevant because contemporary interventional studies in pancreatoduodenectomy have primarily focused on antibiotic selection and microbiological coverage rather than prolonged prophylactic duration.

These findings suggest that biliary contamination is more readily considered in the context of postoperative infection and antibiotic management than as part of POPF risk assessment. This may underestimate its relevance to fistula-related morbidity, as microbiological factors may influence whether a postoperative pancreatic leak remains clinically silent or progresses to CR-POPF.

The strongest clinical support for this view comes from recent studies on perioperative antibiotic prophylaxis in PD. In the randomized trial by D’Angelica et al. [9], piperacillin–tazobactam significantly reduced not only surgical site infections but also clinically relevant POPF compared with cefoxitin in patients undergoing open pancreatoduodenectomy, with the largest benefit seen in patients with preoperative biliary drainage. The microbiological subanalysis by Ellis et al. [5] demonstrated that cefoxitin-resistant biliary organisms—predominantly *Enterococcus* and *Enterobacter* species—were strongly associated with both surgical site infections and clinically relevant POPF in patients receiving cefoxitin, but not in those treated with piperacillin–tazobactam. These findings suggest that inadequate antimicrobial coverage of biliary pathogens may contribute to the progression from biochemical leak to clinically relevant fistula. Observational studies have reported similar associations between positive bile cultures, antibiotic resistance, and fistula-related morbidity, and targeted intraoperative antibiotic adaptation may reduce the adverse effect of biliary contamination on postoperative outcomes [7,13].

A plausible mechanism is that resistant biliary organisms, left uncovered by prophylaxis, may sustain local infection at the anastomosis and drive the progression from biochemical leak to clinically relevant fistula. In intestinal models, *Enterococcus faecalis* activates matrix metalloproteinases, degrades collagen, and promotes anastomotic leak [14,15]. These data are from non-pancreatic settings but point to a link between specific bacterial species and impaired anastomotic healing.

Cephalosporins may not adequately cover the organisms most relevant in this setting. Enterococci are intrinsically resistant, and *Enterobacter cloacae* complex and related Enterobacterales may develop cephalosporin resistance through inducible AmpC β-lactamase production during exposure, despite initial susceptibility [16]. Because these organisms are common in contaminated bile after preoperative drainage, a cephalosporin-based regimen may leave important biliary pathogens uncovered, and prolonging it would not compensate for an inadequate spectrum.

A recent meta-analysis [11] and large-scale real-world data [10] showed reductions in surgical site infections, clinically relevant POPF, and mortality with broad-spectrum penicillin-based prophylaxis, particularly in patients with preoperative biliary drainage. The available evidence therefore supports adequate antimicrobial coverage more clearly than prolonged administration, for which evidence remains limited.

Recent stewardship data place these findings in context. In a pancreatic surgery-specific antimicrobial stewardship program combining preoperative rectal screening for multidrug-resistant organisms with tailored prophylaxis, surgical site infections and antibiotic consumption both decreased, and the association between colonized bile and surgical site infection was no longer detectable after implementation [17,18]. Although these studies did not assess antibiotic selection or duration as determinants of CR-POPF, they demonstrate that antimicrobial management can be tailored to microbiological risk while reducing unnecessary exposure.

McKay and Dixon similarly interpreted the available evidence as supporting a microbiological contribution to the progression from biochemical leak to clinically relevant POPF, particularly after preoperative biliary drainage, and concluded that broad-spectrum penicillin-based prophylaxis should be regarded as the new standard of care [19]. They emphasized, however, that dedicated stewardship programs using preoperative rectal screening and intraoperative bile cultures are needed to refine regimen selection, guide de-escalation and define the appropriate duration. In most contemporary PD populations, and particularly after preoperative biliary drainage, piperacillin–tazobactam remains the empirical regimen with the strongest direct clinical evidence against cephalosporin-based prophylaxis, although local resistance patterns and microbiological information may justify more targeted regimens.

Current guidelines recommend discontinuing surgical antimicrobial prophylaxis at wound closure, as continued administration does not appear to further reduce surgical site infections and may increase the risk of Clostridioides difficile infection and antimicrobial resistance [20,21]. The association between consideration of suspected biliary contamination and longer routine antibiotic duration therefore raises an antimicrobial stewardship concern. However, the survey did not distinguish prolonged prophylaxis from pre-emptive treatment or treatment of suspected infection. Similarly, although adaptation necessarily implied prolongation among respondents using intraoperative-only prophylaxis, the direction of adaptation was not recorded for the remainder. The findings on antibiotic duration should therefore be interpreted in this context. The optimal postoperative antibiotic strategy in patients with biliary contamination is being examined in ongoing randomized trials [22,23].

The findings also have implications for how biliary contamination is incorporated into perioperative risk assessment. Although the risk-assessment and antibiotic-adaptation items assessed distinct constructs and should not be interpreted as directly equivalent measures, the marked difference in reported frequencies is notable: Most surgeons reported adapting antibiotic duration in cases of suspected or proven biliary contamination, but only a minority included it when assessing POPF risk. This suggests that microbiological risk is often handled as an antibiotic management issue, but not as a factor in fistula-related risk assessment. Established POPF risk models focus mainly on pancreatic texture, duct diameter, BMI, and intraoperative factors, and do not explicitly include microbiological variables [24,25,26]. Given the growing evidence linking resistant biliary organisms to infectious morbidity and CR-POPF, especially after preoperative biliary drainage, suspected biliary contamination may deserve more explicit consideration alongside established anatomical and technical risk factors.

This should not be interpreted as support for routine prolongation of antibiotic therapy. The available evidence in PD relates primarily to the choice of agent rather than to the length of administration, particularly in patients with preoperative biliary drainage. Fewer than half of respondents routinely used a ureidopenicillin, indicating that reported antibiotic selection remains highly variable.

### Strengths and Limitations

This study has several strengths. It is based on an international sample of 283 surgeons from 48 countries and addresses a clinically relevant question. Because the survey included separate items on antibiotic class, duration, and duration adjustment, we were able to examine different aspects of antibiotic decision-making rather than relying on a single outcome.

Several limitations should be considered. The survey captures self-reported practice rather than observed patient-level management and may be subject to recall and reporting bias; the cross-sectional design also precludes causal inference. Consideration of biliary contamination was based on a survey response and not on documented bile cultures, preoperative biliary drainage, or other patient-level microbiological data. Moreover, suspected biliary contamination was not formally defined, and AP04 referred to suspected or proven contamination without distinguishing specific clinical triggers or prolonged prophylaxis from pre-emptive treatment or treatment of suspected infection. The survey did not collect resistance patterns, local antibiograms, antibiotic dosing or timing, or patient outcomes, and reported antibiotic class therefore cannot establish microbiological appropriateness.

Only 28 respondents reported considering biliary contamination when assessing POPF risk, limiting the precision of the effect estimates and the ability to detect moderate differences, particularly in antibiotic class selection. A non-significant result in this setting should not be interpreted as evidence of no association. In addition, the 9.9% reporting consideration of biliary contamination in POPF risk assessment and the 69.0% reporting adaptation of antibiotic duration arose from differently worded survey items assessing distinct constructs and with slightly different denominators. They should therefore not be interpreted as directly equivalent measures; any contrast between them is descriptive and should be interpreted qualitatively.

The number of surgeons invited could not be determined, and the study represents a convenience sample; selection bias cannot therefore be excluded, and the reported proportions should not be interpreted as prevalence estimates for the specialty as a whole. Institutional identifiers were incomplete and repeated institutional clusters were sparse, while country-level clustering was not modelled because of the small, exposed group and the sparse and highly unbalanced distribution of respondents across 48 countries. Adjustment was restricted to geographic region and institutional volume, and residual confounding by institutional protocols, local resistance patterns, national guidance, antimicrobial availability, and other unmeasured factors remains possible. Finally, the analyses were not formally prespecified and no multiplicity adjustment was applied to the main analyses. A false-discovery-rate sensitivity analysis did not materially alter the findings, which should nevertheless be considered exploratory and hypothesis-generating.

## 4. Materials and Methods

### 4.1. Study Design and Survey Structure

This study is an exploratory secondary analysis of a previously conducted international survey endorsed by the E-AHPBA. The online survey design and primary descriptive findings have been reported previously [12]. Data were collected between 24 October 2025 and 26 January 2026. The survey was distributed electronically by the E-AHPBA to all its members and additionally through the CALGP (German Chapter of the IHPBA), the Dutch Pancreatic Cancer Group, the ACHBT (French Chapter), the AICEP (Italian Chapter), IHPBA mailing lists, and established professional networks of HPB surgeons. The target population was surgeons performing pancreatoduodenectomy. The questionnaire was piloted by the investigators before distribution and administered using SoSci Survey, with conditional branching so that respondents were shown only items relevant to their previous answers. Because respondents may have been reached through several channels and onward distribution could not be tracked, we cannot determine the number of surgeons invited or calculate a response.

The survey assessed institutional characteristics and technical and perioperative strategies for POPF prevention after pancreatoduodenectomy, including risk assessment, anastomotic technique, drainage, somatostatin analog use, and antibiotic management. A total of 340 survey interviews were recorded, of which 283 were completed (completion rate 83.2%). Participation was anonymous. No technical safeguard against repeated participation was implemented. Completed questionnaires were screened for identical response patterns; none were identified, although repeated participation cannot be excluded with certainty. The complete survey instrument, previously published with the primary report, is also provided as File S1 for completeness. Reporting follows the CROSS checklist (File S2).

The descriptive findings of the survey, including antibiotic class selection, routine antibiotic duration, reported adaptation of duration, and the proportion of respondents considering suspected biliary contamination in POPF risk assessment, have been reported previously [12]. We reproduce these data here only where needed as denominators or for context. The primary report did not examine associations between the risk-assessment and antibiotic items; all associations presented in the current analysis are reported for the first time. The analytical hypotheses were formulated after review of the descriptive survey findings but before these associations were examined. The analysis was not registered or formally prespecified and is therefore considered exploratory. AP02–AP04 were designated as the three principal antibiotic outcomes; threshold analyses, subgroup analyses, the AP02 × AP04 and AP03 × AP04 analyses, and the RA03 analysis were additional exploratory analyses.

### 4.2. Study Population

A total of 283 surgeons from 48 countries completed the survey. Of these, 268 (94.7%) reported using perioperative antibiotics (survey item AP01 = yes). Analyses of antibiotic class selection, routine duration, and duration adaptation (survey items AP02–AP04) were restricted to these participants. The variables used in the principal analyses were complete among these 268 respondents; no item-level missing data were present for RA04 or AP02–AP04. For the adjusted analyses, we grouped these 268 respondents by geographic region: the Northern/Western WHO European Region (*n* = 135), the Southern/Eastern WHO European Region (*n* = 69), and other world regions (*n* = 64). One respondent specified a piperacillin/tazobactam-based regimen in the free-text “other” field as ureidopenicillin use, consistent with the primary report [12].

Institution was recorded as an optional free-text item and was available for 219 of 283 respondents (77.4%), identifying 199 distinct institutions. Fourteen institutions were represented by more than one respondent, accounting for 34 respondents.

### 4.3. Survey Variables and Outcomes

Respondents were grouped according to item RA04, a multiple-response question asking which risk factors influenced decision-making. Those selecting “Suspected biliary contamination” were classified as considering suspected biliary contamination in POPF risk assessment.

Among respondents using perioperative antibiotics (AP01), three outcomes were examined. AP02 asked “Which antibiotics do you typically use for perioperative prophylaxis?” (second-generation cephalosporins, aminopenicillins, ureidopenicillins, or other). AP03 asked “How long do you typically administer intravenous antibiotic prophylaxis?” (intraoperative only, 1–2 days, 3–5 days, or >5 days) and was analyzed as an ordinal outcome. AP04 asked “Do you adapt the duration in case of suspected or proven biliary contamination (e.g., due to preoperative biliary stenting)?” and was analyzed as yes versus no. AP02 and AP03 describe routine practice, whereas AP04 refers specifically to suspected or proven biliary contamination. We considered ureidopenicillin use a marker of broader empirical biliary coverage rather than microbiological appropriateness.

### 4.4. Association Analyses

Respondent characteristics were compared between surgeons who did and did not consider suspected biliary contamination in POPF risk assessment. We then assessed whether consideration of suspected biliary contamination was associated with antibiotic class selection (AP02), routine antibiotic duration (AP03), and adaptation of antibiotic duration (AP04). For AP02, ureidopenicillin use was additionally compared with all other antibiotic classes. Additional analyses examined the relationship between antibiotic class selection and adaptation of duration (AP02 × AP04, Table S1), between routine duration and adaptation of duration (AP03 × AP04, Table S2), and between self-reported risk-adapted management (RA03) and consideration of suspected biliary contamination.

### 4.5. Statistical Analysis

Categorical variables were summarised as counts and percentages. For nominal variables with more than two categories, global associations were assessed using chi-square tests or Fisher–Freeman–Halton exact tests, as appropriate. For 2 × 2 comparisons, Fisher’s exact test was used, and conditional maximum-likelihood odds ratios (ORs) with exact 95% confidence intervals (CIs) were reported. Absolute differences were additionally reported for the principal binary comparisons, and a prevalence ratio was reported for adaptation of antibiotic duration. The association between consideration of suspected biliary contamination and routine antibiotic duration (AP03) was evaluated using ordinal logistic regression with a proportional odds model, reported as a common OR with 95% CI; the proportional odds assumption was assessed using the test of parallel lines and additionally examined using cumulative binary models at all three duration thresholds. An adjusted sensitivity analysis for all three antibiotic outcomes was restricted to geographic region and institutional pancreatoduodenectomy volume, given the small number of respondents considering suspected biliary contamination (Table S3). We did not apply multiplicity adjustment to the main analyses; as a sensitivity analysis, we applied the Benjamini–Hochberg procedure across the three principal antibiotic outcomes to control the false discovery rate. All tests were two-sided, and we considered *p* < 0.05 statistically significant. Analyses were performed using IBM SPSS Statistics, version 29 (IBM Corp., Armonk, NY, USA).

## 5. Conclusions

Despite growing evidence linking biliary contamination to infectious complications and CR-POPF after pancreatoduodenectomy, few surgeons in this international survey reported considering suspected biliary contamination in their POPF risk assessment. Among those who did, consideration of biliary contamination was associated with longer routine antibiotic duration, whereas differences in antibiotic class selection remained uncertain. This is clinically relevant because current evidence provides stronger support for adequate coverage of biliary organisms than for simply extending duration. Whether antibiotic selection or duration influences fistula-related outcomes was not assessed in this survey.

## Figures and Tables

**Table 1:** Respondent characteristics according to consideration of suspected biliary contamination in POPF risk assessment.

Variable	Not Considered (*n* = 255)	Considered (*n* = 28)	*p*
**Institution type**			0.110
Academic	182 (71.4%)	18 (64.3%)	
Public	57 (22.4%)	5 (17.9%)	
Private	16 (6.3%)	5 (17.9%)	
**Annual institutional PD volume**			0.140
≤25	45 (17.6%)	8 (28.6%)	
26–50	80 (31.4%)	12 (42.9%)	
51–75	52 (20.4%)	4 (14.3%)	
>75	78 (30.6%)	4 (14.3%)	
**Personal annual PD volume**			0.546
≤15	76 (29.8%)	8 (28.6%)	
16–30	113 (44.3%)	15 (53.6%)	
31–50	43 (16.9%)	2 (7.1%)	
>50	23 (9.0%)	3 (10.7%)	
**Years in practice**			0.086
≤5	29 (11.4%)	0 (0.0%)	
6–10	43 (16.9%)	2 (7.1%)	
11–15	40 (15.7%)	5 (17.9%)	
>15	143 (56.1%)	21 (75.0%)	
**Risk-adapted management**			0.646
Yes	193 (75.7%)	20 (71.4%)	
No	62 (24.3%)	8 (28.6%)	

Data are *n* (%). PD—pancreatoduodenectomy. Consideration was defined as selection of “suspected biliary contamination” among factors influencing POPF risk assessment (item RA04_05). Global comparisons used Fisher–Freeman–Halton exact tests for r × c tables and Fisher’s exact test for 2 × 2 tables.

**Table 2:** Association between consideration of suspected biliary contamination in risk assessment and antibiotic strategy.

Antibiotic Strategy Outcome	Not Considered (*n* = 240)	Considered (*n* = 28)	OR (95% CI)	*p*
**Antibiotic class selection (AP02)**				
Global comparison				0.192
Ureidopenicillins	105 (43.8%)	15 (53.6%)	1.48 (0.63–3.55)	0.422
2nd-gen. cephalosporins	89 (37.1%)	6 (21.4%)		
Aminopenicillins	28 (11.7%)	6 (21.4%)		
Other	18 (7.5%)	1 (3.6%)		
**Antibiotic duration (AP03)**				
Overall association (ordinal model)			2.29 (1.14–4.59)	0.020
Intraoperative only	114 (47.5%)	7 (25.0%)		
1–2 days	49 (20.4%)	5 (17.9%)		
3–5 days	61 (25.4%)	15 (53.6%)		
>5 days	16 (6.7%)	1 (3.6%)		
**Adaptation of antibiotic duration for suspected or proven biliary contamination (AP04)**				
Yes	160 (66.7%)	25 (89.3%)	4.15 (1.21–22.11)	0.016
No	80 (33.3%)	3 (10.7%)		

Analyses were restricted to 268 respondents reporting perioperative antibiotic use (item AP01 = yes), of whom 28 considered and 240 did not consider suspected biliary contamination in risk assessment (item RA04_05). Data are presented as *n* (%) unless otherwise stated. OR, odds ratio; CI, confidence interval. AP02 was analyzed using the Fisher–Freeman–Halton exact test; AP03 using ordinal logistic regression; 2 × 2 comparisons using Fisher’s exact test with conditional maximum-likelihood odds ratios and exact 95% confidence intervals.

## Data Availability

Data are contained within the article or Supplementary Materials. The original contributions presented in this study are included in the article/Supplementary Materials. Further inquiries can be directed to the corresponding author.

## Supplementary Materials

The following supporting information can be downloaded at: https://www.mdpi.com/article/10.3390/antibiotics15090922/s1, Table S1: Association between antibiotic class selection and adaptation of antibiotic duration (AP02 × AP04); Table S2: Association between routine antibiotic duration and adaptation of antibiotic duration (AP03 × AP04); Table S3: Association between consideration of suspected biliary contamination in POPF risk assessment and the three principal antibiotic outcomes: unadjusted and adjusted analyses; File S1: Full survey instrument (questionnaire); File S2: Checklist for Reporting of Survey Studies (CROSS).

## Abbreviations

The following abbreviations are used in this manuscript:

PD	Pancreatoduodenectomy
POPF	Postoperative pancreatic fistula
CR-POPF	Clinically relevant postoperative pancreatic fistula
E-AHPBA	European-African Hepato-Pancreato-Biliary Association
IHPBA	International Hepato-Pancreato-Biliary Association
BMI	Body mass index
OR	Odds ratio
CI	Confidence interval
