# Supplementary material for: Association Between Surgeons’ Consideration of Suspected Biliary Contamination and Reported Antibiotic Duration After Pancreatoduodenectomy: An Exploratory Secondary Analysis of an International Survey
Source: Antibiotics (Basel). 2026 Sep 17;15(9):922. doi: 10.3390/antibiotics15090922 (PMC13603344; doi:10.3390/antibiotics15090922)
Supplement: Supplementary file 1 [file antibiotics-15-00922-s001.zip › Supplementary Material File S1_Questionnaire.pdf]

Dear Colleagues,

we warmly invite you to participate in this international survey, **endorsed by the European-African Hepato-Pancreato-Biliary Association (E-AHPBA)**. The purpose of this project is to assess current technical and perioperative strategies used to prevent postoperative pancreatic fistula (POPF) following pancreatoduodenectomy (PD).

Despite continuous advances in pancreatic surgery, POPF remains a major cause of postoperative morbidity and mortality. Multiple techniques and adjunctive measures have been proposed to mitigate this risk — including variations in pancreatic anastomosis, stent use, antibiotic prophylaxis, and somatostatin analogues. However, practices differ widely, and high-level evidence remains limited.

This survey aims to capture these variations, identify areas of consensus and controversy, and lay the foundation for future collaborative research.

Your participation is **anonymous**, and completing the survey will take **approximately 10 minutes**. The results will be shared with the E-AHPBA community and may serve as a basis for subsequent prospective trials.

We sincerely appreciate your valuable time and expertise.

With kind regards,
Dr. Michael Hoffmann – University Hospital Augsburg, Germany
Dr. David Pinto – University Hospital Augsburg, Germany
Prof. Katharina Beyer – University Hospital Augsburg, Germany
Prof. Matthias Anthuber (emeritus) – University Hospital Augsburg, Germany

1. Institutional and Surgeon Information

IA01

1. Country:

IA02

2. Name of Medical Institution (optional):

IA03

3. Type of Medical Institution:

- ◯ Academic Hospital
- ◯ Public Hospital
- ◯ Private Hospital

IA04

4. Annual number of pancreatoduodenectomies at your institution:

- ◯ ≤25
- ◯ 26–50
- ◯ 51–75
- ◯ >75

IA05

5. Personal number of pancreatoduodenectomies/year:

- ○ ≤15
- ○ 16–30
- ○ 30–50
- ○ >50

IA06

6. How many years have you been practicing as a surgeon?

- ○ ≤5
- ○ 6–10
- ○ 11–15
- ○ >15

IA07

7. Are you a sub-specialized HPB surgeon?

- ○ Yes
- ○ No

2. Risk Assessment Practices

RA01

8. Do you perform an intraoperative risk assessment for POPF (e.g., using the Fistula Risk Score or evaluation of gland texture and duct diameter)?

- ○ Always
- ○ Often
- ○ Rarely
- ○ Never

1 Active Filter(s)

Filter RA01/F1
If any of the following options is selected: **4**
Then hide question/text **RA02** placed later in the questionnaire

RA02

9. If yes, which score or tool do you typically use?

- ◯ Fistula Risk Score
- ◯ ISGPS classification for intraoperative risk stratification
- ◯ Subjective clinical judgment only
- ◯ Other (please specify)

RA03

10. Do you adjust your intra- and perioperative management (e.g., use of pancreatic stents, somatostatin analogues, reinforcement techniques, drain strategies) based on the estimated POPF risk?

- ◯ Yes
- ◯ No

RA04

11. Which of the following risk factors influence your decision-making? (Select all that apply):

- ☐ Pancreatic texture
- ☐ Duct diameter
- ☐ Suspected biliary contamination
- ☐ Blood loss
- ☐ BMI
- ☐ Other (please specify): ____________

RA05

12. Would you consider performing a total pancreatectomy in patients with very high risk for POPF (e.g., FRS 8-10 or ISGPS type D)?

- ◯ Yes
- ◯ No

3. Pancreatic Anastomosis

13. What is your preferred type of pancreatoenteric anastomosis? PA01

- ◯ Pancreaticojejunostomy
- ◯ Pancreaticogastrostomy
- ◯ Other (please specify): ______

14. If Pancreaticojejunostomy is used, what is your preferred technique? PA02

- ◯ Blumgart (duct-to-mucosa with transpancreatic U-sutures)
- ◯ Cattell-Warren (duct-to-mucosa with outer seromuscular layer)
- ◯ Pure duct-to-mucosa (single-layer, no outer seromuscular row)
- ◯ Invagination (whole stump invaginated into jejunum)
- ◯ Other (please specify): ______

15. Do you use reinforcement techniques at the anastomosis site (e.g., mesh, omentum)? PA03

- ◯ Yes
- ◯ No

1 Active Filter(s)

Filter PA03/F1
If any of the following options is selected: **1**
Then display question/text **PA04** placed later in the questionnaire (otherwise hide)

PA04

16. If yes, which of the following techniques do you use? (Select all that apply):

- ☐ Omental wrapping
- ☐ Falciform or round ligament wrapping
- ☐ Polyglycolic acid mesh or patch
- ☐ Fibrin glue or other sealants
- ☐ Other (please specify): ____

PA05

17. Do you modify your anastomotic technique depending on the patient's risk profile?

- ○ Yes
- ○ No

4. Pancreatic Stents

PS01

18. Do you place a pancreatic duct stent during reconstruction?

- ○ Always
- ○ Often
- ○ Rarely
- ○ Never

5 Active Filter(s)

Filter PS01/F1
If any of the following options is selected: **4**
Then hide question/text **PS02** placed later in the questionnaire

Filter PS01/F2
If any of the following options is selected: **4**

Then hide question/text **PS03** placed later in the questionnaire

Filter PS01/F3
If any of the following options is selected: **4**
Then hide question/text **PS04** placed later in the questionnaire

Filter PS01/F4
If any of the following options is selected: **4**
Then hide question/text **PS05** placed later in the questionnaire

Filter PS01/F5
If any of the following options is selected: **1**
Then hide question/text **PS05** placed later in the questionnaire

PS02

19. If yes, what type of stent do you use?

- ◯ Internal
- ◯ External
- ◯ Depends on case

PS03

20. If you place a pancreatic duct stent, what kind of material or device do you typically use?

- ◯ Internal plastic stent originally designed for ERCP
- ◯ Cut segment of an infant feeding tube
- ◯ Cut central venous catheter (CVC)
- ◯ Cut pancreatic drainage catheter
- ◯ Bioabsorbable stent (e.g. PDS-based)
- ◯ Long catheter externally tunneled through the jejunal and abdominal wall
- ◯ Other (please specify): ______

PS04

21. Please describe your standard stent technique or product more specifically (optional):

______

PS05

22. In which clinical risk settings do you place a pancreatic stent? (Select all that apply):

- ☐ Soft pancreatic texture
- ☐ Small duct diameter
- ☐ Other (please specify): ______

5. Antibiotic Prophylaxis

23. Do you use perioperative antibiotics?

AP01

- Yes
- No

3 Active Filter(s)

Filter AP01/F1
If any of the following options is selected: **2**
Then hide question/text **AP02** placed later in the questionnaire

Filter AP01/F2
If any of the following options is selected: **2**
Then hide question/text **AP03** placed later in the questionnaire

Filter AP01/F3
If any of the following options is selected: **2**
Then hide question/text **AP04** placed later in the questionnaire

AP02

24. Which antibiotics do you typically use for perioperative prophylaxis?

- ◯ 2nd generation cephalosporins (e.g., cefuroxime; ± metronidazole)
- ◯ Aminopenicillins (e.g., ampicillin, amoxicillin; ± beta-lactamase inhibitors)
- ◯ Ureidopenicillins (e.g., piperacillin; ± beta-lactamase inhibitors)
- ◯ Other (please specify): ____

AP03

25. How long do you typically administer intravenous antibiotic prophylaxis?

- ◯ Only intraoperative prophylaxis (shortly before incision ± intraoperative re-dosing) -
- ◯ 1-2 days
- ◯ 3-5 days
- ◯ >5 days

AP04

26. Do you adapt the duration in case of suspected or proven biliary contamination (e.g., due to preoperative biliary stenting)?

- ◯ Yes
- ◯ No

6. Somatostatin Analogues

SA01

27. Do you use somatostatin analogues to prevent POPF?

- ◯ Always
- ◯ Often
- ◯ Rarely
- ◯ Never

3 Active Filter(s)

Filter SA01/F1
If any of the following options is selected: **4**
Then hide question/text **SA02** placed later in the questionnaire

Filter SA01/F2
If any of the following options is selected: **4**
Then hide question/text **SA03** placed later in the questionnaire

Filter SA01/F3
If any of the following options is selected: **4**
Then hide question/text **SA04** placed later in the questionnaire

28. If yes, which agent do you use? SA02

- Octreotide
- Pasireotide
- Lanreotide
- Somatostatin (continuous infusion)

29. What is your standard duration of somatostatin analogue therapy? SA03

- 1–2 days
- 3–5 days
- >5 days

30. Do you limit somatostatin analogue use to high-risk cases? SA04

- Yes
- No

7. Drainage Practices

31. How many intra-abdominal drains do you usually place after PD? DP01

- None
- One drain
- Two drains
- More than two drains

4 Active Filter(s)

Filter DP01/F1
If any of the following options is selected: **1**
Then hide question/text **DP02** placed later in the questionnaire

Filter DP01/F2
If any of the following options is selected: **1**
Then hide question/text **DP03** placed later in the questionnaire

Filter DP01/F3
If any of the following options is selected: **1**
Then hide question/text **DP06** placed later in the questionnaire

Filter DP01/F4
If any of the following options is selected: **1**
Then hide question/text **DP07** placed later in the questionnaire

DP02

32. What type of intra-abdominal drains do you typically use after PD?

- ◯ Easy Flow drain (passive, soft silicone)
- ◯ Robinson drain (passive, rigid PVC tube)
- ◯ Blake drain – used with active suction
- ◯ Blake drain – used passively (without suction)
- ◯ Jackson-Pratt drain – used with active suction
- ◯ Jackson-Pratt drain – used passively (without suction)
- ◯ Salem-Sump drain (double-lumen, may be used for irrigation)
- ◯ Other (please specify): ______

DP03

33. Do you routinely measure drain fluid amylase or lipase postoperatively to monitor for POPF?

- ◯ Yes – daily until drain removal
- ◯ Yes – on specific days (e.g. POD 1, 3, 5)
- ◯ Yes – but only if clinically indicated
- ◯ No – not part of routine practice

DP04

34. Do you use perianastomotic irrigation drains after PD in high-risk patients?

- ◯ Always
- ◯ Often
- ◯ Rarely
- ◯ Never

1 Active Filter(s)

Filter DP04/F1
If any of the following options is selected: **4**
Then hide question/text **DP05** placed later in the questionnaire

DP05

35. If you use irrigation drains: When do you typically initiate flushing?

- ○ Immediately postoperative
- ○ On postoperative day 1 or 2
- ○ Only if drain fluid is lipase-positive
- ○ Other (please specify): ____

DP06

36. Which factors influence the timing of drain removal? (Select all that apply):

- ☐ Drain amylase or lipase
- ☐ Output volume
- ☐ Clinical signs
- ☐ Institutional Protocol
- ☐ Other (please specify): ____

DP07

37. What is your usual timing for prophylactic drain removal after PD, provided the clinical course is uneventful and drain fluid characteristics are favorable?

- ○ POD 1-3
- ○ POD 4-5
- ○ POD 6-7
- ○ Later than POD 7

FC01

38. Do you have any additional comments on the prevention of POPF or the content of this survey?

Thank you for completing this questionnaire!

We sincerely appreciate the time and thought you have invested in completing this survey. Your responses will contribute to a better understanding of current practice and help guide future collaborative research within the HPB community.

Your answers were submitted, you may close the browser window or tab now.

With kind regards,

The POPF Survey Team
European-African Hepato-Pancreato-Biliary Association (E-AHPBA)
University Hospital Augsburg, Germany
