# Supplementary material for: Association Between Surgeons’ Consideration of Suspected Biliary Contamination and Reported Antibiotic Duration After Pancreatoduodenectomy: An Exploratory Secondary Analysis of an International Survey
Source: Antibiotics (Basel). 2026 Sep 17;15(9):922. doi: 10.3390/antibiotics15090922 (PMC13603344; doi:10.3390/antibiotics15090922)
Supplement: Supplementary file 1 [file antibiotics-15-00922-s001.zip › Supplementary Material File S2_Cross.pdf]

File S2. Checklist for Reporting of Survey Studies (CROSS)

Completed for: Association Between Surgeons' Consideration of Suspected Biliary Contamination and Reported Antibiotic Duration After Pancreatoduodenectomy: An Exploratory Secondary Analysis of an International Survey.

Section/topic	Item	CROSS item description	Reported in
**Title and abstract**			
Title and abstract	1a	State the word "survey" along with a commonly used term in title or abstract to introduce the study's design.	Title; Abstract (Methods)
	1b	Provide an informative summary in the abstract, covering background, objectives, methods, findings/results, interpretation/discussion, and conclusions.	Abstract
**Introduction**			
Background	2	Provide a background about the rationale of study, what has been previously done, and why this survey is needed.	Section 1
Purpose/aim	3	Identify specific purposes, aims, goals, or objectives of the study.	Section 1, final two paragraphs
**Methods**			
Study design	4	Specify the study design in the methods section with a commonly used term.	Section 4.1 — exploratory secondary analysis of a cross-sectional international survey
Data collection methods	5a	Describe the questionnaire (e.g., number of sections, number of questions, number and names of instruments used).	Section 4.1; File S1 (complete questionnaire)
	5b	Describe all questionnaire instruments used to measure particular concepts. Report target population, validity and reliability information, scoring/classification procedure, and reference links.	Section 4.1 and File S1 — target population and complete survey instrument
	5c	Provide information on pretesting of the questionnaire, if performed. Report method, number of times pre-tested, number and demographics of participants, and similarity to the sample population.	Section 4.1 — questionnaire piloted by the investigators before distribution
	5d	Questionnaire should be fully provided (in the article, appendices, or online supplement).	File S1 (complete questionnaire including response options and filter logic)
Sample characteristics	6a	Describe the study population (background, locations, eligibility criteria for inclusion, exclusion criteria).	Section 4.1 — surgeons performing pancreatoduodenectomy, internationally; analyses restricted to respondents reporting perioperative antibiotic use (AP01 = yes)
	6b	Describe the sampling techniques used. Specify locations of sample participants whenever clustered sampling was applied.	Section 4.1 — convenience sample recruited through professional societies and networks
	6c	Provide information on sample size, along with details of sample size calculation.	Section 4.1 — no a priori sample-size calculation was performed; the secondary analysis included all 283 completed questionnaires from the existing survey dataset
	6d	Describe how representative the sample is of the study population.	Section 3.1 — convenience sample, geographically unbalanced; not representative
Survey administration	7a	Provide information on modes of questionnaire administration, including type and number of contacts and the location where the survey was conducted.	Section 4.1 — online survey (SoSci Survey); distributed by E-AHPBA, IHPBA, CALGP, Dutch Pancreatic Cancer Group, ACHBT, AICEP and professional networks
	7b	Provide information on the survey's time frame, such as periods of recruitment, exposure, and follow-up days.	Section 4.1 — 24 October 2025 to 26 January 2026
	7c	For web-based surveys, provide approaches to prevent multiple participation of participants.	Section 4.1 — no technical safeguard against repeated participation; completed questionnaires screened for identical response patterns, none identified.
Study preparation	8	Describe any preparation process before conducting the survey (e.g., interviewers' training process, advertising the survey).	Section 4.1 — questionnaire development and piloting by the investigators; endorsement and review by the E-AHPBA Scientific Committee

Ethical considerations	9a	Provide information on ethical approval, including informed consent, IRB approval, Helsinki declaration, and GCP declaration as appropriate.	No formal ethics approval required; consent implied by voluntary participation
	9b	Provide information about survey anonymity and confidentiality and describe mechanisms used to protect unauthorized access.	Section 4.1 and Data Availability Statement — anonymous participation; no direct personal identifiers were collected; survey data were collected using SoSci Survey
Statistical analysis	10a	Describe statistical methods and analytical approach. Report the statistical software used.	Section 4.5
	10b	Report any modification of variables used in the analysis, along with reference if available.	Sections 4.3 and 4.5 — one free-text antibiotic response reclassified as ureidopenicillin; country grouped into three regions (WHO European Region); institutional volume entered as an ordinal score
	10c	Report details about how missing data was handled, including rate of missing items, missing data mechanism, and methods used.	Sections 4.1–4.2 — variables used in the principal analyses were complete among the 268 respondents included in the antibiotic analyses; no item-level missing data were present for RA04 or AP02–AP04. Institution was an optional free-text item and was available for 219 of 283 respondents.
	10d	State how non-response error was addressed.	Section 3.1 — not formally addressed; the number of surgeons invited could not be determined and non-respondents could not be characterised
	10e	For longitudinal surveys, state how loss to follow-up was addressed.	Not applicable — cross-sectional design
	10f	Indicate whether weighting of items or propensity scores were used to adjust for non-representativeness.	No weighting or propensity-score adjustment for non-representativeness was applied
	10g	Describe any sensitivity analysis conducted.	Section 4.5; Sections 2.4 and 2.6; Tables S2 and S3 — cumulative binary duration thresholds and adjusted models
**Results**			
Respondent characteristics	11a	Report numbers of individuals at each stage of the study. Consider using a flow diagram.	Sections 4.1 and 2.1 — 340 interviews recorded, 283 completed, 268 reporting perioperative antibiotic use, and 28 reporting consideration of suspected biliary contamination.
	11b	Provide reasons for non-participation at each stage, if possible.	Not available; reasons for non-participation or discontinuation were not recorded.
	11c	Report response rate, present the definition or formula used.	Section 4.1 — a response rate cannot be calculated as the number of surgeons invited is unknown; completion rate 283/340 = 83.2% (completed questionnaires divided by recorded interviews)
	11d	Provide information to define how unique visitors are determined. Report number of unique visitors along with relevant proportions.	Not determinable; unique visitors were not tracked.
Descriptive results	12	Provide characteristics of study participants, as well as information on potential confounders and assessed outcomes.	Section 2.2; Table 1
Main findings	13a	Give unadjusted estimates and, if applicable, confounder-adjusted estimates along with 95% confidence intervals and p values.	Sections 2.3–2.6; Table 2; Tables S1–S3
	13b	For multivariable analysis, provide information on the model building process, model fit statistics, and model assumptions.	Section 4.5; Section 2.4 (test of parallel lines); Table S3 (covariate selection); no additional global model-fit statistics reported
	13c	Provide details about any sensitivity analysis performed.	Sections 2.4 and 2.6; Tables S2 and S3
**Discussion**			
Limitations	14	Discuss the limitations of the study, considering sources of potential biases and imprecisions.	Section 3.1
Interpretations	15	Give a cautious overall interpretation of results, based on potential biases and imprecisions, and suggest areas for future research.	Section 3

Generalizability	16	Discuss the external validity of the results.	Section 3.1
**Other sections**			
Role of funding source	17	State whether any funding organization has had any roles in the survey's design, implementation, and analysis.	Funding statement
Conflict of interest	18	Declare any potential conflict of interest.	Conflicts of Interest statement
Acknowledgements	19	Provide names of organizations/persons that are acknowledged along with their contribution to the research.	Acknowledgments
