# Supplementary material for: Association Between Surgeons’ Consideration of Suspected Biliary Contamination and Reported Antibiotic Duration After Pancreatoduodenectomy: An Exploratory Secondary Analysis of an International Survey
Source: Antibiotics (Basel). 2026 Sep 17;15(9):922. doi: 10.3390/antibiotics15090922 (PMC13603344; doi:10.3390/antibiotics15090922)
Supplement: Supplementary file 1 [file antibiotics-15-00922-s001.zip › Supplementary_Table S1_AP02_AP04_.pdf]

Supplementary Table S1. Association between antibiotic class selection and adaptation of antibiotic duration (AP02 × AP04)

Antibiotic class (AP02)	Adaptation (n = 185)	No adaptation (n = 83)
2nd-generation cephalosporins	66 (35.7%)	29 (34.9%)
Aminopenicillins	27 (14.6%)	7 (8.4%)
Ureidopenicillins	77 (41.6%)	43 (51.8%)
Other	15 (8.1%)	4 (4.8%)

Data are n (%). Analyses restricted to 268 respondents reporting perioperative antibiotic use (AP01 = yes).

Global comparison performed using Pearson's chi-square test ($p = 0.270$).
