# Supplementary material for: Association Between Surgeons’ Consideration of Suspected Biliary Contamination and Reported Antibiotic Duration After Pancreatoduodenectomy: An Exploratory Secondary Analysis of an International Survey
Source: Antibiotics (Basel). 2026 Sep 17;15(9):922. doi: 10.3390/antibiotics15090922 (PMC13603344; doi:10.3390/antibiotics15090922)
Supplement: Supplementary file 1 [file antibiotics-15-00922-s001.zip › Supplementary_Table S2_AP03_AP04.pdf]

Supplementary Table S2. Association between routine antibiotic duration and adaptation of antibiotic duration (AP03 × AP04)

Reported routine duration (AP03)	Total	Adaptation reported	No adaptation reported
Only intraoperative prophylaxis	121	74 (61.2%)	47 (38.8%)
1–2 days	54	34 (63.0%)	20 (37.0%)
3–5 days	76	62 (81.6%)	14 (18.4%)
>5 days	17	15 (88.2%)	2 (11.8%)
**Total**	**268**	**185 (69.0%)**	**83 (31.0%)**

Data are n (row %). Analyses restricted to 268 respondents reporting perioperative antibiotic use (AP01 = yes).

Global comparison performed using Pearson's chi-square test ($\chi^2 = 12.97$, df = 3, $p = 0.005$).

Among the 121 respondents reporting intraoperative-only prophylaxis, 74 (61.2%) reported adapting duration in case of suspected or proven biliary contamination. In this subgroup, adaptation can only correspond to prolongation, as no shorter regimen exists.
