# Supplementary material for: Association Between Surgeons’ Consideration of Suspected Biliary Contamination and Reported Antibiotic Duration After Pancreatoduodenectomy: An Exploratory Secondary Analysis of an International Survey
Source: Antibiotics (Basel). 2026 Sep 17;15(9):922. doi: 10.3390/antibiotics15090922 (PMC13603344; doi:10.3390/antibiotics15090922)
Supplement: Supplementary file 1 [file antibiotics-15-00922-s001.zip › Supplementary_Table S3_adjusted.pdf]

Supplementary Table S3. Association between consideration of suspected biliary contamination in POPF risk assessment and the three principal antibiotic outcomes: unadjusted and adjusted analyses

Outcome and model	OR (95% CI)	p value
**Reported antibiotic duration (AP03, ordinal)**		
Unadjusted	2.29 (1.14–4.59)	0.020
Adjusted for region	2.30 (1.14–4.63)	0.019
Adjusted for region and institutional volume	2.09 (1.03–4.25)	0.042
**Reported adaptation of duration (AP04)**		
Unadjusted	4.17 (1.22–14.22)	0.023
Adjusted for region	4.34 (1.26–14.92)	0.020
Adjusted for region and institutional volume	3.51 (1.01–12.19)	0.048
**Reported ureidopenicillin use (AP02)**		
Unadjusted	1.48 (0.68–3.25)	0.325
Adjusted for region	1.48 (0.67–3.25)	0.331
Adjusted for region and institutional volume	1.86 (0.82–4.19)	0.135

Analyses restricted to 268 respondents reporting perioperative antibiotic use (AP01 = yes), of whom 28 reported considering suspected biliary contamination when assessing POPF risk. Odds ratios refer to consideration of suspected biliary contamination.

AP03 was analysed using an ordinal (proportional odds) model; AP02 and AP04 using binary logistic regression. All estimates in this table, including the unadjusted models, are regression-based maximum-likelihood odds ratios with Wald confidence intervals and Wald p values, so that the three models are directly comparable. The corresponding unadjusted results in the main text are based on Fisher's exact test with conditional maximum-likelihood odds ratios and exact confidence intervals, and the p values therefore differ slightly (AP04: 0.016 exact vs. 0.023 Wald; AP02: 0.422 exact vs. 0.325 Wald).

Given the small number of respondents in the consideration group, the adjusted sensitivity analysis was deliberately restricted to geographic region and institutional pancreatoduodenectomy volume, selected as the two contextual factors considered most directly relevant to local antibiotic practice. Institutional volume was entered as an ordinal score across the four volume categories (≤25, 26–50, 51–75, >75 per year). Personal surgeon volume and years in practice were not additionally included to preserve model parsimony.

Region groups were defined using the WHO European Region. Northern/Western WHO European Region (n = 135): Germany 52, France 27, United Kingdom 15, Netherlands 14, Belgium 7, Denmark 4, Finland 4, Norway 4, Sweden 4, Austria 2, Switzerland 2. Southern/Eastern WHO European Region (n = 69): Italy 27, Spain 13, Greece 10, Türkiye 5, Hungary 3, Slovenia 2, Romania 2, Lithuania 2, Azerbaijan 1, Israel 1, Portugal 1, Slovakia 1, Ukraine 1. Other world regions (n = 64): United States 26, South Africa 5, India 4, Japan 4, Australia 4, Argentina 3, Canada 2, Colombia 2, Mexico 2, and one respondent each from Algeria, Brazil, Cameroon, China, Ecuador, Ghana, Hong Kong SAR, Mongolia, Nepal, New Zealand, Philippines and Venezuela.
